# Towards a theoretical understanding of false positives in DNA motif finding

**DOI:** 10.1186/1471-2105-13-151

**Published:** 2012-06-27

**Authors:** Amin Zia, Alan M Moses

**Affiliations:** 1 Department of Cell & Systems Biology, University of Toronto, 25 Willcocks Street, Toronto, ON, M5S 3B2, Canada

## Abstract

**Background:**

Detection of false-positive motifs is one of the main causes of low performance in *de novo* DNA motif-finding methods. Despite the substantial algorithm development effort in this area, recent comprehensive benchmark studies revealed that the performance of DNA motif-finders leaves room for improvement in realistic scenarios.

**Results:**

Using large-deviations theory, we derive a remarkably simple relationship that describes the dependence of false positives on dataset size for the one-occurrence per sequence motif-finding problem. As expected, we predict that false-positives can be reduced by decreasing the sequence length or by adding more sequences to the dataset. Interestingly, we find that the false-positive strength depends more strongly on the number of sequences in the dataset than it does on the sequence length, but that the dependence on the number of sequences diminishes, after which adding more sequences does not reduce the false-positive rate significantly. We compare our theoretical predictions by applying four popular motif-finding algorithms that solve the one-occurrence-per-sequence problem (MEME, the Gibbs Sampler, Weeder, and GIMSAN) to simulated data that contain no motifs. We find that the dependence of false positives detected by these softwares on the motif-finding parameters is similar to that predicted by our formula.

**Conclusions:**

We quantify the relationship between the sequence search space and motif-finding false-positives. Based on the simple formula we derive, we provide a number of intuitive rules of thumb that may be used to enhance motif-finding results in practice. Our results provide a theoretical advance in an important problem in computational biology.

## Background

Because binding of sequence specific transcription factors to their recognition sites in non-coding DNA is an important step in the control of gene expression, the development of computational methods to identify transcription factor binding motifs in non-coding DNA has received much attention in computational biology [1]. The low information content of transcription factor binding motifs implies difficulty for computational analyses. For example, given a known binding motif, identification of bona fide examples is always plagued by false positives - the so-called Futility Theorem [1].

An even more challenging computational problem is the *de novo* identification of transcription factor binding motifs (so-called motif-finding), for which there are many available tools (for tutorials on different methods see [2,3] and references therein). Despite the substantial algorithm development effort in this area, most recent comprehensive benchmark studies [4-6] revealed that the performance of DNA motif-finders leaves room for improvement in realistic scenarios, where known transcription factor binding sites have been planted in test sequence sets.

One explanation for these observations could be that the low information content of DNA binding sites places limits on this problem as well - an extension of the Futility Theorem [1] to the *de novo* motif-finding problem. This has led to development of a large number of motif finding algorithms that attempt to include additional data in the motif-finding problem to improve the signal to noise ratio. For example, including quantitative high-throughput gene expression or binding measurements [7-10], phylogenetic information [11-14], transcription factor structural class [15,16], nucleosome-positioning information [17], local sequence composition and GC content [18], improved background models [19-21], or different motif-finding models [21] have all been shown to improve motif-finding results in practice.

Here we argue that ‘false positive motifs’, i.e., patterns similar to typical biological motifs, may be likely to arise due to the statistical nature of large sequence data sets. In other words, when the dataset is large enough, motifs with strength similar to real transcription factor binding motifs begin to occur by chance. Consistent with this idea, it is frequently observed that DNA motif-finders identify seemingly strong candidate motifs, even when randomly chosen sequences are provided as the input. This issue has been previously recognized [22] in the so-called “twilight zone search”- a motif-finding scenario where the probability of observing random motifs with higher score than real motifs is non-negligible. It was shown that the detection of false-positives, particularly in the twilight zone, is inevitable [22].

The prevalence of such false positive motifs in DNA motif-finding has led to substantial research to assess the statistical significance of motifs. It is important to distinguish three distinct types of research in this area. The first aims to calculate of p-values for matches to a given motif (e.g., [23,24]) and is not directly relevant to the motif-finding problem considered here. The second aims to calculate the p-value of a motif itself, which is an ungapped multiple alignment [25,26]), while the third concerns the statistical significance of a motif identified through a ‘motif-finding’ procedure (e.g., [22]).

The second and third types of research are closely related, and were both treated in the seminal work of Hertz & Stormo [27], which used large deviations theory to approximate the motif distribution, and provided algorithms to approximate the p-value of the ungapped alignment. Recent work has led to highly efficient algorithms based on Fast Fourier transforms (FFT) to compute these p-values [26] and given a motif of interest (or ungapped multiple alignment) it is now possible to obtain a p-value.

Hertz & Stormo [27] also proposed a method to assign significance to motifs identified in a motif-finding procedure by assuming that the motif finder can explore the entire space of possible motifs and select the most significant one. The p-value for the identified motif is then ‘corrected’ for the number of possible motifs considered and converted to an E-value that is defined as the expected number of random motifs that would have information context at least as high as the given motif [27]. Therefore, the false-positive rate is closely related to the motif p-value and can be reduced if an accurate p-value is available. However, the E-value suggested by Hertz & Stormo does not always provide a useful measure of significance particularly in the cases where in it desired to detect weak motifs, i.e. when there is a reasonable chance of finding motifs of similar strength in random sequences [28].

In practice, significance of motifs identified through motif-finding is often obtained through simulations (e.g. [21]) where the motif finder is run on random sequences either drawn from or generated based on the dataset, and significance is assessed using the strength of motifs identified in these random datasets (‘false positives’) as the null distribution. While it is assumed that this distribution can be approximated by Gumbel distribution [29], it been shown empirically that it fits very well to 3-parameter Gamma distributions [30] and when significance is assessed using this null-distribution, the false positive rate can be significantly reduced [18]. While simulation-based methods are very useful to assess the significance of a motif-finding result, they do not provide insight as to how the false-positive rate changes as a function of the motif-finding problem parameters and therefore cannot be used to design experiments to avoid false-positives.

Here, we obtain a remarkably simple analytical relationship between the size of the sequence search space and the strength of the false-positive motifs (we provide a definition for the strength of a motif below). In particular, we use Sanov’s theorem [31] to derive a bound on the p-value of motifs with a given strength. We then use this to relate the sample size at which less than one false-positive is expected and the strength of the false-positives (when they do occur) to the parameters of the motif-finding problem.

Since we have considered the underlying statistics of the one-occurrence-per-sequence motif-finding problem, our results should apply to any motif-finding method that attempts to solve this problem. We confirmed this with softwares that implement different optimization approaches, MEME [32,33] which uses Expectation-Maximization, GIMSAN [34,35] and the original Gibbs Sampler [36-38] (the latter two being MCMC methods). Interestingly, when we compared the false positives produced by a fourth software, Weeder [39,40], which uses combinatorial algorithm based on suffix trees to build the motifs and does not restrict the problem to one-occurrence-per-sequence, we also found similar statistics for the false positives, suggesting that our theoretical analysis may be robust to motif-finding assumptions. Because of the simplicity of the analytic relationship between dataset size and false positive strength, we present simple rules of thumb that we believe will be useful in study design, as well as aid in interpreting the results of *de novo* motif finders.

## Results

### A bound on the p-value of a motif

**Figure 1:**
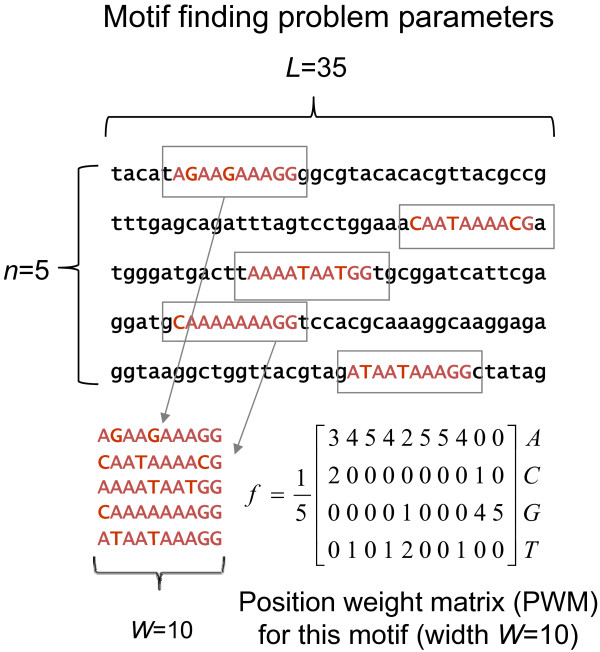
**DNA motif finding problem parameters.** In this example, *n* = 5 sequences of length *L* = 35 are used to detect a motif of width *W* = 10. The corresponding PWM, *f*, is also shown. Note that each sequence has exactly one occurrence of the motif.

We first consider the problem of assigning a p-value to a motif (or ungapped multiple alignment). The patterns in DNA sequence families (called motifs) can be represented by position weight matrices (PWMs), in which each column specifies the distribution of the DNA letters [41,42] (for a tutorial on motif-finding see [3]). We refer to the set of *n* sub-sequences of width *W* aligned together as a motif (see Figure 1 and Methods for definition of motif finding problem parameters). We define the PWM, *f,* for a motif as a matrix where each column contains the parameters of a categorical distribution (see Figure 1 for details). The categorical distribution is defined as a probability distribution that describes the result of a single trial where one of K possible outcomes is randomly selected (e.g. K = 4 for DNA). The categorical distribution is commonly referred to imprecisely as the “multinomial distribution”, which describes the result of *n* trials. Unless otherwise stated all probability distributions on nucleotides throughout the text are categorical distributions. The difference between the distribution of the motif represented by the position weight matrix, *f*, and the background distribution, *g,* is measured using the Kullback–Leibler (KL) divergence [31] also known as the biological information content [3,18], defined as follows:

(1) $$D(f,g)=\sum_{j=1}^{W}\sum_{k=\left\{A,T,C,G\right\}}^{}f_{\mathrm{jk}}\log_{2}\frac{f_{\mathrm{jk}}}{g_{k}}$$

where *f*_*jk*_ is the relative frequency of base *k* in column *j* of the motif, and *g*_*k*_ is the background distribution of base *k* (e.g. the genomic distribution of nucleotides). Throughout the text we use the strength of a motif, its specificity, and its information content, interchangeably to refer to *D*(*fg*). According to the Law of Large Numbers [31], the distribution, *f*, of any motif generated by sampling from a background distribution, *g*, should be arbitrarily close to *g* (in the probability sense). Therefore, observing a motif with *f* that is significantly different from *g* is extremely unlikely.

Under the null hypothesis (of randomly generated sequences) a PWM with *f* significantly different from *g* is considered as a rare event and far from expectation. We use the large-deviations theory, in particular Sanov’s theorem [31], to measure an upper bound on the probability of these rare events. Consider a motif with PWM *f* that is diverged from the background *g* by *D*(*fg*) (we commonly state that the motif has a strength *D*(*fg*)). We define the p-value of the motif as the probability of observing a motif by chance that is diverged from the background (or has a strength) at least by *D*(*fg*) (see Additional file 1: Appendix for precise mathematical definition of p-value). We prove that that p-value is upper-bounded as follows:

(2) $$p-value_{\mathrm{motif}}\leq {\left(n+1\right)}^{W(\left|A\right|-1)}2^{-nD(f,g)}$$

where *A* is the alphabet (A,C,G,T for DNA) and |*A*| is the cardinality of the set *A*, e.g. |*A*| = 4 for DNA sequences. Please see the Appendix ( Additional file 1: Appendix) for the proof of this theorem. We note that the bound in (2) is not tight; depending on *f* it can be significantly loose and agree poorly with the accurate p-values (see Additional file 1: Appendix). Nevertheless, the qualitative behaviour of this p-value as the parameters are varied does seem to agree with the behaviour of p-values obtained using more accurate methods ( Additional file 2: Figure S1).

### Theoretical bounds on false positives in de novo motif finding

We now turn to our main focus, which is the problem of false positives in *de novo* motif finding. We now consider a set of *n* sequences of length *L* generated according to a background distribution *g* of nucleotides. We assume that we have an ideal motif-finder that will identify the *n* sub-sequences (one from each sequence of length *L*) that when aligned will give the strongest motif, i.e., the motif with the largest difference from the background as measured by *D*(*f*,*g*). This is referred to as the ‘one-occurrence-per-sequence’ (oops) motif finding problem (see Figure 1 and Methods for definition of motif finding problem parameters).

Our main theoretical results are as follows. If the sequence length (*L*) is less than the following bound, the expected number of motifs that occur by chance with strength equal or greater than *D*(*f*,*g*) is less than 1.

(3) $$L<(W-1)+\frac{2^{D(f,g)}}{{\left(n+1\right)}^{W(\left|A\right|-1)/n}}$$

Here |*A*| is the cardinality of the set *A*, e.g. |*A*| = 4 for DNA sequences. In other words, the expected number of false positives is less than 1 when the inequality (3) holds.

Furthermore, when one or more motifs are expected to occur by chance with strength *D*(*f*,*g*) greater than some threshold D*, the threshold D* is less than the following bound:

(4) $$D^{*}\leq \log_{2}L+(|A|-1)W\frac{\log_{2}(n+1)}{n}$$

Thus, our theory predicts that when false positives occur, their strength will depend differently on each of the motif finding parameters *L, W* and *n* (see Figure 1 for explanation of these).

To obtain these results, we have followed Hertz & Stormo, and assumed that the ideal motif-finder has tested all (*L - w +* 1)^*n*^ possible motifs. Please see Appendix A ( Additional file 1: Appendix) for the proof of these results.

### False positives are predicted to arise in realistic motif-finding scenarios

**Figure 2:**
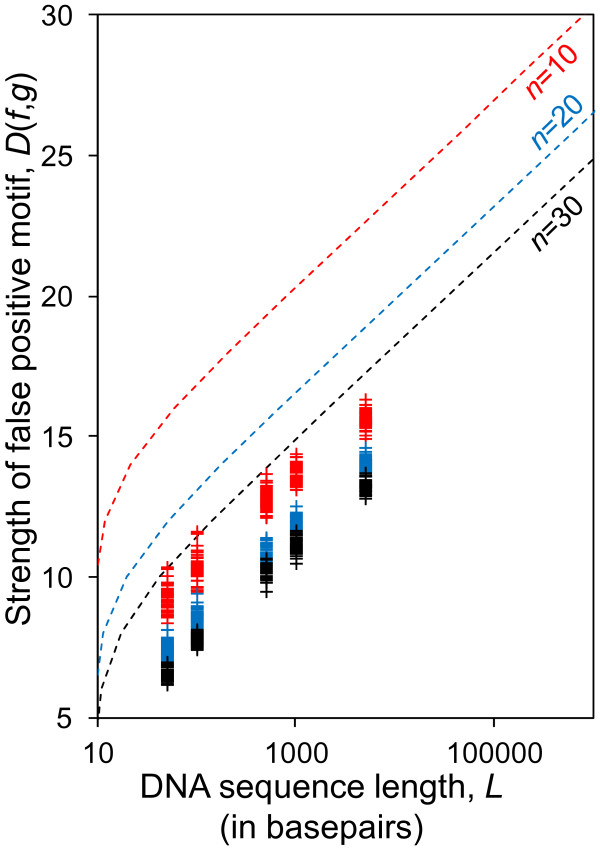
**Theoretical bound on sequence length compared with results from MEME.** Theoretical bound sequence length, *L*, at which less than one false-positive motif with information content *D*(*f*,*g*) is expected (solid lines) compared to experimental results of MEME (crosses) for motif width *W* = 10. The region of the plot above and to the left of the traces represents the parameter space in which less than one false positive is expected. The results are for three different numbers of sequences, *n* = {10,20,30} indicated by red, blue and black symbols and traces, respectively.

We next sought to test whether the typical dataset sizes used for DNA motif-finding are likely to produce false positives according the formula above. Figure 2 shows *L* as a function of motif information content, *D*(*f*,*g*)*,* for DNA sequences with typical motif-finding parameters including the number of sequences in the dataset *n* = {10, 20, 30} and the motif width *W* = 10. The graph illustrates the length of the sequences, *L*, below which less than 1 false-positive motifs with strength *D*(*f*,*g*) are expected to occur by chance (similar graphs for different sets of parameters are shown in Additional file 2: Figure S2).

**Figure 3:**
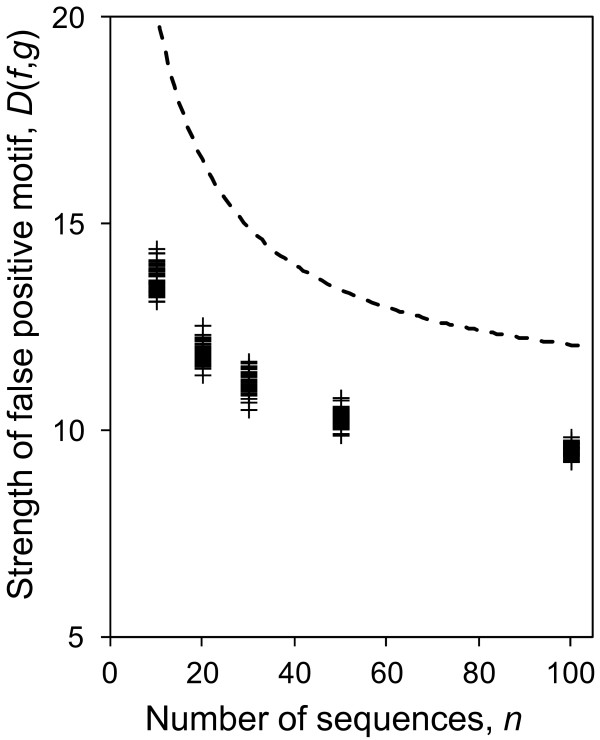
**The relationship between false-positive information content and the number of sequences.** The figure shows the theoretical upper bound on the information content threshold, D*, when one or more false-positive motif is expected to be observed in a dataset as function of the number of sequences, *n* (dashed line) compared to the strength of false-positive motifs detected by MEME (crosses). For both cases *n* is chosen from *n* = {10,20,30,50,100} and the parameters *L* = 1000 and *W* = 10 are fixed. The strength of motifs detected by MEME is consistent with the strength of motifs predicted to occur by chance for the given sample size.

We note that the bound on false positives (predicted by Eq. 3) depends more strongly on *n* than on *L*. As an example, for motifs with *W* = 10 (Figure 2), a threefold increase of *n* (while keeping *L* constant) reduces *D*(*f*,*g*) by the same amount as if *L* were decreased by 2 orders of magnitude (while keeping *n* unchanged). However, the dependency of false-positive strength on *n* decreases as *n* becomes large (Eq. 4). This predicts that the effect of adding more sequences on the reduction of false-positives diminishes when *n* becomes large (Figure 3).

**Figure 4:**
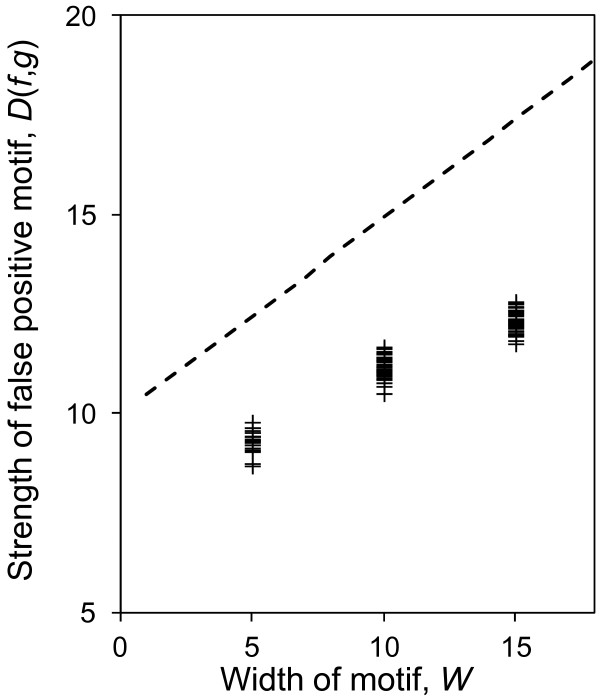
**The relationship between false-positive information content and the motif width.** The figure shows the theoretical upper bound on the information content threshold, D*, when one or more false-positive motif is expected to be observed in a dataset as function of motif width *W* (dashed line) compared to the strength of false-positive motifs detected by MEME (crosses). For both cases *W* is chosen from *n* = {5, 10, 15} and the parameters *L* = 1000 and *n* = 30 are fixed. The strength of motifs detected by MEME is consistent with the strength of motifs predicted to occur by chance for the given sample size.

Finally, the upper bound on false-positive strength threshold, D*, is approximately linear in *W* (Eq. 4, Figure 4). Therefore, for a given motif strength (i.e. motifs with a given divergence), detecting real motifs with smaller widths is less prone to false-positives. We note that the width (*W*) of a real transcription factor binding motif is set by the biophysical interaction of the transcription factor with DNA, and is therefore not a parameter that we can control in experimental design. Real motifs with larger width tend to have greater information content, and therefore are usually easier to detect in motif-finding experiments.

### MEME, the Gibbs sampler, GIMSAN, and weeder performance is qualitatively consistent with the theoretical expectations

To confirm our theoretical results, we conducted a series of experiments with four popular motif finding softwares: MEME [32,33] and the Gibbs Sampler [36-38], Weeder [39,40], and GIMSAN [34,35] (see Methods for details of the experimental setup). As input to these softwares, we generated random datasets (according to a uniform nucleotide distribution, see Methods) where we specified the length of sequences (*L*) and the number of sequences (*n*). Because the DNA sequences are randomly generated, we can be sure that any discovered motifs are false positives.

We first performed extensive simulations with the MEME software because it allows the user to specify the parameters of the motif-finding problem, such as the width of the motif and the one-occurrence-per-sequence assumption. This allows us to directly compare our theoretical predictions of the dependence of false positives on the motif finding parameters to the observed false positives (Eq. 4). The results from MEME qualitatively follow the theoretical prediction (Figures 2, 3, 4 and Additional file 2: Figure S2) as they do not appear in the regions of the plots where the expected number of false positives is less than 1.

**Figure 5:**
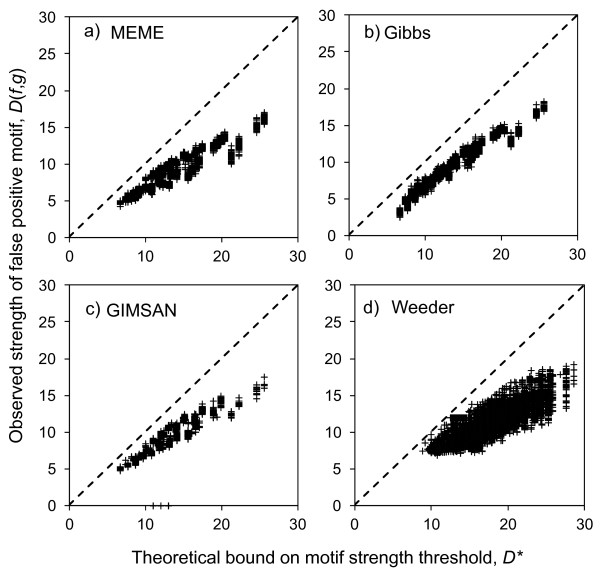
**Comparison of theoretical bound and observed false-positive motif strengths.** The strengths of observed false positive motifs identified by **a**) MEME, **b**) the Gibbs Sampler, **c**) GIMSAN and **d**) Weeder show reasonable accordance with our theoretical bound. Each cross represents one false positive motif, while the dashed line represents ‘*y = x*’ where the theoretical bound equals the observations. The results include motifs for *L* = 50,100,500,1000 and *n* = 10,20,30,50,100. For a), b) and c) the results are for motifs of *W* = 5, 10 and 15, while for d) the results are for *W* = 6,8,10 and 12.

Since our theory is based only on the statistics of random sequences, it should be applicable to any motif finder that solves the one-occurrence-per-sequence motif finding problem, regardless of the algorithm used for optimization. To test this, we compared the strength of each false positive motif discovered by MEME and the Gibbs Sampler to the bound predicted by Eq. 4. For both MEME and the Gibbs Sampler, we found similar agreement between the observed false positives and the theoretical bound (R^2^ > 0.85, Figure 5-a,b).

We also tested GIMSAN because of its unique approach for computing p-values based on the estimation of the null distribution for motifs. We asked GIMSAN to find motifs with widths (*W* = 5,10,15) in our randomly generated datasets (see Methods for experimental details). We considered only motifs with p-value less than 0.01. The strength of motifs detected by GIMSAN is also consistent with our theoretical bound (R^2^ = 0.83, Figure 5c).

We note that most *de novo* motif-finding algorithms do not allow the user to control the number of occurrences of the motif in the sequence dataset. For example, Weeder is a combinatorial algorithm based on suffix trees that implements an efficient search algorithm for finding similar sub-sequences in the dataset in order to build a consensus and ultimately motifs. Because Weeder was among the best performing algorithms, in terms of false-positive rate, in a benchmark comparison [4], we sought to test whether it would also produce false positives, and if so, how they would deviate from the theoretical bound made based on the one-occurrence-per-sequence assumption.

Because Weeder does not allow the user to specify the width of the motif or the number of motif instances that each sequence will contain, we simply ran it repeatedly on random sequence sets of various sizes and identified false positive motifs (See Methods for more details). To compare the strength of the false positive motifs to the predicted bound on strength of these motifs based on our theoretical results, we defined *'n'* in Eq. 4 above to be the actual number of sequences in the input set in which Weeder identified a motif, and required that at least 5 sequences were included in the motif. Figure 5d shows the comparison of the predicted and observed false positive strengths for each motif identified by Weeder. Interestingly, the strength of the false positive motifs identified by Weeder also shows reasonable accordance with our predictions (R^2^ = 0.60). That the Weeder results show such good agreement with our predictions is somewhat surprising, as Weeder violates the assumptions we made in deriving Eq. 2. This suggests that our theoretical results may be quite robust to the assumptions made in the motif finding procedure (see Discussion).

For all four motif-finders, the false positives identified tend to be weaker than the theory predicts (Figure 5a-d), which is consistent with Eq. 2 giving an upper bound on the p-value, which leads to the upper bound on false positive strength in Eq. 4. Taken together, these data strongly support our hypothesis that false positives are due in part to large size of the motif-finding search space.

## Discussion

We used large-deviations theory to approximate the relationship between false positives and the parameters of the one-occurrence per sequence de novo DNA motif-finding problem. A similar approach has been previously proposed to quantify the so-called twilight zone [22] in terms of the parameters of the motif-finding problem including the dataset size, and our work can be regarded as an extension of that work to the more general ‘matrix’ or probabilistic representation of motifs. However, the previous work did not reveal the surprisingly simple relationship between false positives and the motif-finding parameters. Nevertheless, both our work and the previous findings suggest that false positives are likely due at least in part to the statistics of random sequences, rather than any algorithmic or biological reason.

We note that the situation we considered is where each position in the DNA sequence is considered to be drawn from a background distribution *g* independently and identically. However, real genomic sequences do not follow this simple assumption [43-46]. DNA bases at adjacent positions are correlated, likely due to the complex mutational processes that create them. Interestingly, this means that our theoretical and simulation results represent a ‘best case scenario’. In real, correlated genomes, even stronger false positive motifs will be identified by an ideal motif-finder.

### Simple rules of thumb for DNA motif finding

To reduce the false-positive strength in experimental design, it is generally desired to move towards weaker false-positive motifs. The theoretical predictions provide intuition about how to adjust motif-finding parameters to reduce the strengths of motifs that are due to chance (using Eq. 4 or using the curves in Figures 2, 3 and 4). We have the following rules of thumb for this purpose:

· As it is intuitively expected, it is generally preferred to use shorter sequences (when it is biologically plausible) to avoid false-positives.

· Adding more sequences to the dataset reduces the false-positive rate considerably (e.g. using 30 sequences compared to 10 reduces the false-positive motif strengths by more than 6 bits (~25%) for *W* = 10, see Figure 3). This effect, however, diminished for larger *n* (e.g. increasing *n* from 30 to 50 has only 2 bits reduction in false positive motif strengths. This suggests that in order to reduce false-positive rate in motif finding, only a “sufficient” number of sequences is needed (in this case ~30).

· The dependency of false-positives (the strength of false-positive motifs) on *L* is weaker than dependency on *n*. Therefore, using many sequences (but not too many) is generally preferred to using shorter sequences.

· For a given information content, the detection of motifs with smaller width is less prone to false-positives. Therefore, to avoid false positives, it is generally preferred to choose the smallest possible width that adequately summarizes the biological motif.

### Examples of applications

In using the theoretical results in Eq. 3 or the graphs in Figure 2, it is generally desired to move towards weaker false positive motifs (towards the bottom on the graphs). To illustrate this we chose the Zfp423 and the TATA-box motifs from the Jaspar database [47] with *D*(*fg*) = 17.93 and *D*(*fg*) = 10.20, respectively (both with a width of *W* = 15).

**Figure 6:**
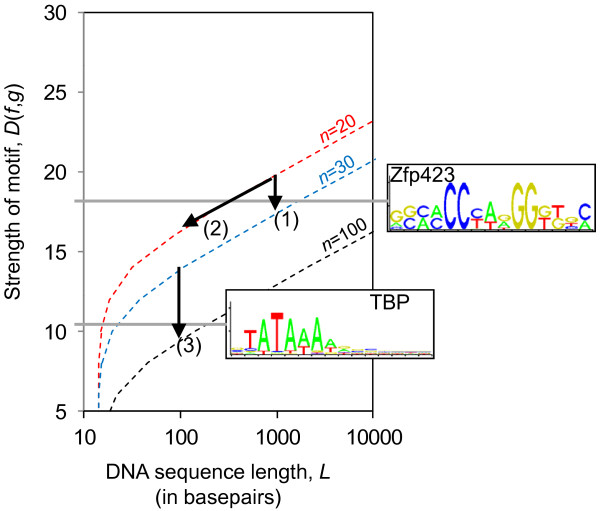
**Examples of applications of the theoretical results.** Two real motifs of width *W* = 15 from the JASPAR database (ZFP423 and TATA-box, TBP) are used to illustrate the application of the theoretical predictions. For a strong motif like Zfp423, increasing the number of sequences, *n*, from 20 to 30 (arrow 1) or reducing the sequence length from 1000 to 100 (arrow 2) could sufficiently reduce the bound so that the real motif is found in a region where the expected number of false positives is less than 1. For a weak motif like TBP, detection in sequences of length 100 might still be prone to false positives, so instead a large increase in the number of sequences, *n*, (arrow 3) is needed.

It can be seen from Figure 6 that real motifs as strong as ZFP423 in *n* = 20 sequences of length *L* = 1000 will be below the theoretical traces, and will therefore be expected to be buried among false-positives. To avoid this situation, one can reduce *L* (along Arrow-2) or add more sequences (along Arrow-1) to the dataset. Similarly, it would be very difficult to identify a weak motif such as the TATA-box motif in a set of 30 sequences, even with length *L* = 100, because it is well below the bound where less than 1 false positive is expected. Since using shorter sequences is unlikely, one can increase the number of sequences to *n* = 100 (along Arrow-3) so that the motif is above the bound. Alternatively, trimming all but the core bases of the TATA-box is equivalent to moving along the theoretical curve from *W* = 15 to *W* = 5, and reduces the false-positive bound enough to detect this motif (data not shown).

### Comparison of false positives from different motif finders

To test whether our results were applicable beyond the one-occurrence per sequence setting, in addition to MEME and the Gibbs Sampler, we tested Weeder, a non-probabilistic motif-finder that implements a consensus-based search. We found that the theoretical relationship held quite well for the false-positives produced by Weeder, suggesting that the simple formula we obtained will be quite generally applicable, or that heuristic post-processing steps in Weeder (implemented by the so-called “advisor” program) to reduce the false-positives (by removing the highest scored motifs that do not qualify a redundancy criteria, see [40] for detail) tend to approximate the one-occurrence per sequence constraint.

Regardless of their generality, our theoretical results quantify the limit to how well we can expect even the ideal motif-finder to perform. This will be useful to future benchmarking studies, so they can take into account whether the ‘real’ motif in test cases is strong enough to be distinguished from false positives that spontaneously arise.

## Conclusions

We have derived a remarkably simple formula to describe the relationship between false positive strength and dataset size in the one-occurrence per sequence DNA motif finding problem, and confirmed it using simulations. We conclude that false positives in *de novo* DNA motif finding may result in part because of statistical properties of random DNA sequences, rather than any weaknesses in specific algorithms.

## Methods

### Simulations

In each experiment, we generated a set of *n* = {10,20,30,50,100} sequences with length *L* = {50, 100, 500, 1000} drawn from a uniform background distribution *g* = [0.25 0.25 0.25 0.25]. For each particular *n* and *L*, we repeated the experiments for many Monte-Carlo runs (so there are multiple datasets with the same *n* and *L* and therefore many possible false positive motifs for each set of parameters).

We presented each dataset as input to the softwares. For each detected motif, we computed the information content or divergence, *D(f,g)*, using the PWMs or frequency matrices reported. Since the input to these programs was sets of random sequences, all detected motifs are false-positives. We then compared the false-positives detected with the theoretical predictions.

Particular notes for each software are as follows:

MEME: We ran MEME using OOPS model (one occurrence per sequence) using parameter (−m oops) and restricted MEME to generate only one motif (the most significant) with widths *W* = {5,10,15} (using -w parameter). We ran MEME on 50 random datasets for each *n*, *L* and *W* combination, except for *n* = 20, *L* = 50, *W* = 10 where we obtained 100, yielding a total of 3050 false positive motifs

Gibbs Sampler: we used the “site sampler” model that restricts the software to include in the PWM only one occurrence of the motif in each sequence and with widths *W* = {5,10,15}. We ran the gibbs sampler on 50 random datasets for each *n*, *L* and *W* combination, yielding a total of 3000 false positive motifs.

GIMSAN: we used the OOPS model and considered motifs with widths *W* = {5,10,15}. For each experiment, we used the same set of sequences to compute the background distribution to increase the chance of software for rejecting false-positives. We ran GIMSAN and obtained 11100 motifs. We then rejected any motifs with a p-value (that is provided by GIMSAN) larger than 0.01, yielding 2216 false positive motifs.

WEEDER: We ran Weeder on the random datasets using the “large” parameter. Because Weeder does not allow the user to specify the width of the motif (*W*) or the number of motif instances that each sequence will contain, we simply ran it 29108 times on random sequence sets of various sizes. We then parsed out detected motifs with widths *W* = {6, 8, 10, 12}. To compare the strength of the false positive motifs to the predicted strength of these motifs based on our theoretical results for Weeder, we defined *'n'* in Eq. 3 above to be the actual number of sequences in the input set in which Weeder identified a motif, and removed any motifs with fewer than 5 sequences included, yielding 18700 false positive motifs.

## Competing interests

The authors declare that they have no competing interests.

## Authors’ contributions

AMM and AZ designed the study. AZ performed all research. AMM supervised the research. AMM and AZ wrote the paper. All authors read and approved the final manuscript.

## Supplementary Material

Additional file 1 **Appendix. Proof of the main theorem**[3,31,41,42,48]**].** (PDF 346 kb) (PDF 306 kb) Click here for file

Additional file 2 **Figure S1**. The comparison between bound on the p-value (Eq. 2) and the p-value computed by an FFT-based method. Figure S2. Theoretical bound on sequence length compared with MEME results. Figure S3. Theoretical bound on sequence length compared with GIMSAN results. (PDF 225 kb) Click here for file
