# Supplementary material for: Towards a theoretical understanding of false positives in DNA motif finding
Source: BMC Bioinformatics. 2012 Jun 27;13:151. doi: 10.1186/1471-2105-13-151 (PMC3436861; doi:10.1186/1471-2105-13-151)
Supplement: Additional file 1 — Appendix. Proof of the main theorem[3,31,41,42,48]]. (PDF 346 kb) (PDF 306 kb) [file 1471-2105-13-151-S1.pdf]

Appendix: Proof of the main theorem
Towards a theoretical understanding of false positives in DNA motif finding

Amin Zia and Alan M. Moses

1. Overview

Motif-finding methods search for short stretches of *similar* DNA bases referred to as motifs. We describe the structure of a motif statistically by the relative frequency of the DNA bases in the motif and compare these frequencies with a random background distribution. We refer to the difference between the composition of the bases in the motif and the background distribution as the *strength* or the *specificity* of motif, interchangeably.

Now suppose that the sequences are generated according to a background distribution and therefore do not carry any biologically relevant motif. In this case, any motif detected by motif-finding algorithms is a false-positive. The main theorem proved here gives an approximate size for the dataset for which the detection of such fasle-positive motifs (with a given width and specificity) becomes quite probable.

To prove the main theorem, we use a number of definitions and lemmas from classical information theory and statistics to compute an upper-bound on the p-value of any given motif (based on its width and specificity). The lemmas given here are mainly adopted from [31] with minor modifications. Once the bound on the p-value is derived, we multiply it by the number of possible motifs in the dataset to derive an upper-bound on the number of motifs expected to be observed in the dataset. We use this bound to prove the main theorem.

It is important to note that the results presented here apply to any motif-finding scenario no matter what algorithm is used. In fact we argue that false-positives happen because of the intrinsic randomness residing in the sequences: if the size of the data set is sufficiently large, false-positives with any arbitrary specificity can be observed by chance.

The outline of the proofs is as follows:

- We assume that a motif-finder can explore all possible motifs in a set of sequences randomly generated according to a background distribution. The background distribution is commonly considered as the genome-wide frequency of bases (e.g. a uniform distribution).
- According to the Law of Large Numbers [31], it is extremely unlikely (i.e. it is a rare event) that any motif in the dataset has bases with frequencies significantly different than the background. We therefore use the theory of large deviation to compute the probability of observing these events.
- By adding up the probabilities of all such rare events, i.e. the probability of observing motifs with given strength or higher, we compute an upper bound on the *p-value* of the motif.
- We then use the *p-value* and the number of motifs explored by the motif-finder to compute the expected number of motifs at a given strength.

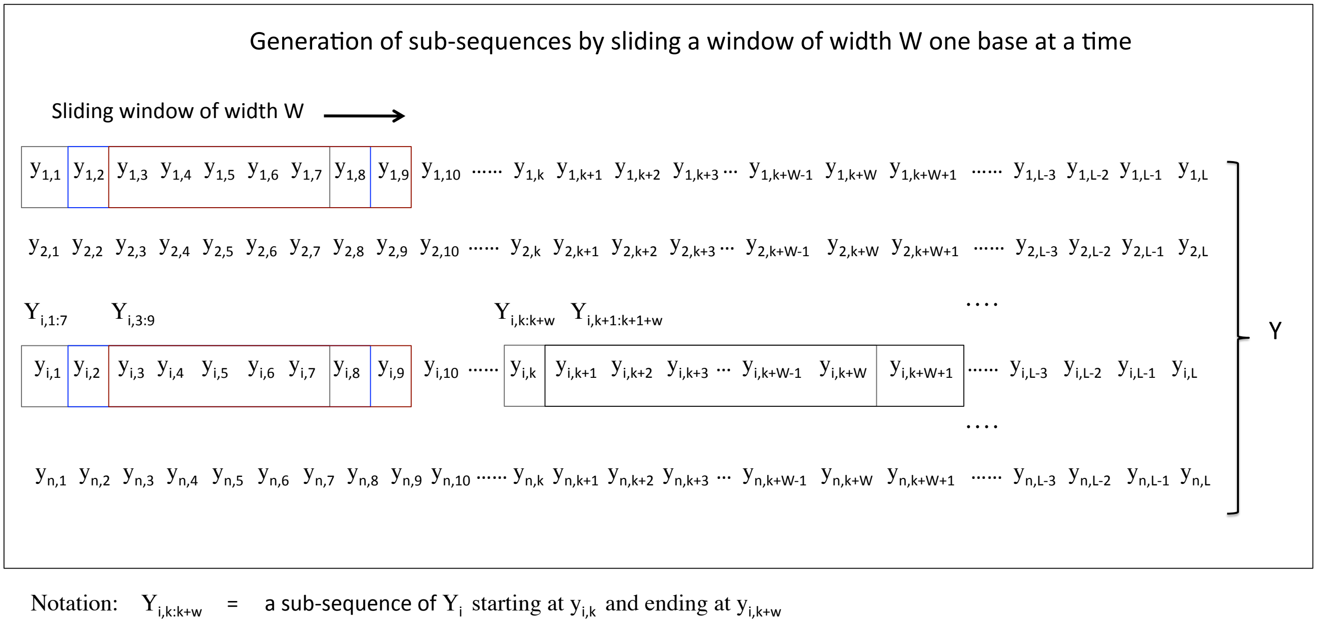

Fig. 1. All sub-sequences of length W are generated by sliding a window of width W over each row of Y, one base at a time.

1.1. *Notations*

We denote by $\mathcal{A} = \{A, C, T, G\}$ the set of DNA bases, which has cardinality 4, i.e. $|\mathcal{A}| = 4$. All theoretical derivations provided here are also valid for other alphabets including the amino-acids for which $|\mathcal{A}| = 20$.

We denote by L and n the length of the sequences and the number of sequences in the dataset, respectively. Also we denote by W the width or size of the motif.

The position weight matrix (PWM) of a motif is a matrix with W columns corresponding to W positions in the motif and 4 rows corresponding to bases $\{A, T, C, G\}$. We represent the PWM with f or h throughout. We define a PWM precisely in the following. The background distribution, with W columns and 4 rows is represented by g. The divergence (defined below) or statistical difference between the motif's PWM and g is measured by the Kullback-Leibrer [31] distance denoted by $D(f, g)$.

We represent matrices or vectors by upper-case variables: Y and X. The upper-case variables with a single index refer to rows of matrices, e.g. Y_i represents the ith row of Y. All vectors are row vectors. When we need to refer to a part of the row of a matrix, we use the convention $Y_{i,k:k+W}$ that represent the vector of W elements (from column k to column $k+W$) of the ith row of Y (see for example Fig. 1). Each single base in the dataset is denoted by lower-case variables, e.g. x and y and indexed by the row and column position in the matrix or the vector: $y_{i,j}$ is the base at the jth column of the ith row of Y.

For any matrix Y, we denote by Y^T the *transpose* of a matrix.

2. Preliminaries

3. Motif finding problem set up

Let us denote by $Y = [Y_1,\ Y_2,\ \dots\ , Y_n]^T$ the set of n sequences with length L used in typical motif-finding problems:

$$Y = \begin{bmatrix} y_{1,1} & y_{1,2} & y_{1,3} & \cdots & y_{1,L-1} & y_{1,L} \\ y_{2,1} & y_{2,2} & y_{2,3} & \cdots & y_{2,L-1} & y_{2,L} \\ \cdots & \cdots & \cdots & \cdots & \cdots & \cdots \\ y_{n,1} & y_{n,2} & y_{n,3} & \cdots & y_{n,L-1} & y_{n,L} \end{bmatrix} \quad (1)$$

where e.g. Y_i is the ith row with L DNA bases.

Motif finding algorithms seek to find a set of over or under-represented short sub-sequences of width W in rows of Y. In order to account for all possible sub-sequences in rows of Y, we slide a window of length W over each row, Y_i, from left to right and shifting one base at a time (see Fig. 1) to generate all possible sub-sequences. As examples, the first three sub-sequences of Y_1 as well as the two consecutive sub-sequences of Y_i, denoted by $Y_{i,k:k+W}$ and $Y_{i,k+1:k+W+1}$, are shown in Fig. 1.

Note that there are $(L - W + 1)$ sub-sequences of length W in Y_i.

Now suppose that we choose one sub-sequence from each row Y_i. This results in n sub-sequences (since Y has n rows). We arrange these sub-sequences in a matrix form, as shown in the following, and refer to it as a motif X (see Fig. 2):

$$X = \begin{bmatrix} x_{1,1} & x_{1,2} & x_{1,3} & \cdots & x_{1,W-1} & x_{1,W} \\ x_{2,1} & x_{2,2} & x_{2,3} & \cdots & x_{2,W-1} & x_{2,W} \\ \cdots & \cdots & \cdots & \cdots & \cdots & \cdots \\ x_{n,1} & x_{n,2} & x_{n,3} & \cdots & x_{n,W-1} & x_{n,W} \end{bmatrix} \quad (2)$$

This motif arrangement is based on the "one-occurrence-per-sequence" model in motif finding where each sequence Y_i contributes one and only one sub-sequence to motifs.

We denote by $\mathcal{X}$ the set of all motifs X obtained by this method and refer to it as the *dataset* throughout:

$$\mathcal{X} = \Big\{ X : X_{i,j} \in \{A, T, C, G\};\ i = 1, ..., n, j = 1, ...W;\ \ X_i \text{ chosen from } Y_i \text{ by the sliding window} \Big\} \quad (3)$$

Each row of Y, e.g. Y_i, consists of $(L - W + 1)$ overlapping sub-sequences of width W. There are n rows in Y. Therefore, the size of the dataset formed by all possible combinations of sub-sequences is:

$$|\mathcal{X}| = (L - W + 1)^n. \quad (4)$$

3.1. *Statistical representation of motifs*

The search for statistically significant motifs, in essence, involves finding $X \in \mathcal{X}$ that is distributed significantly differently from a background distribution g. To do so, we represent the

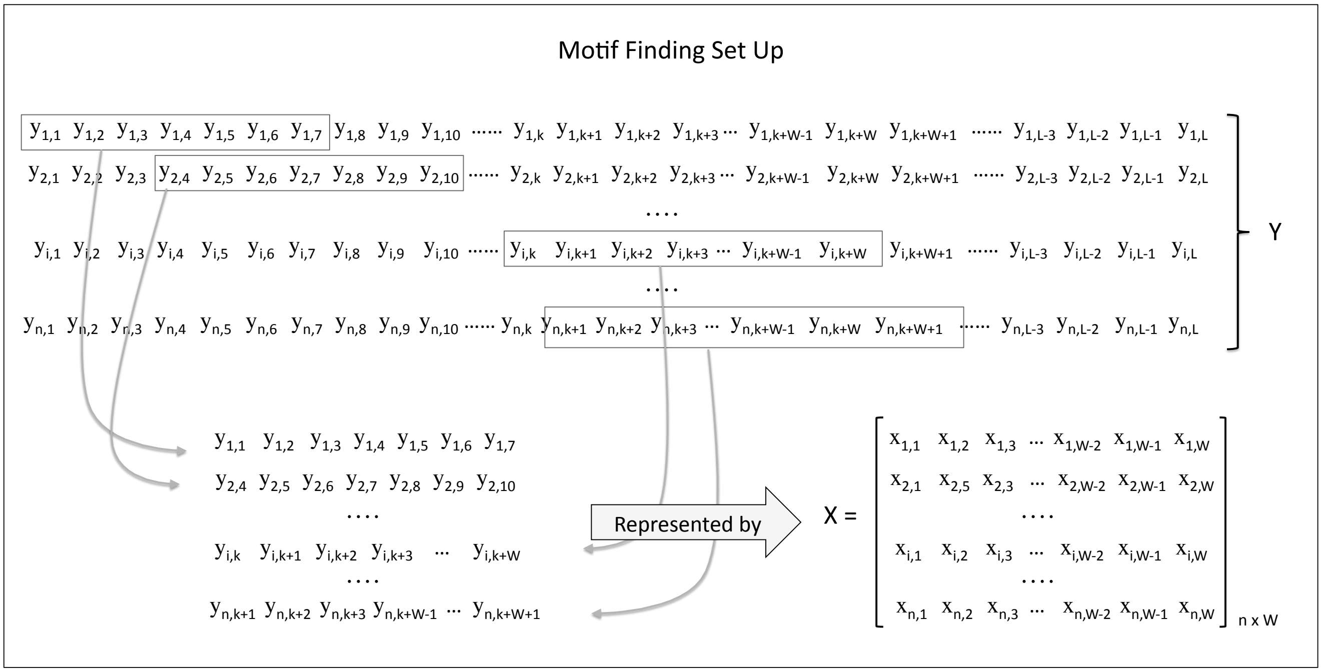

Fig. 2. According to the "one-occurrence-per-sequence" (OOPS) model in motif finding, one and only one sub-sequence from each row of Y is used to form a motif. We represent any of such motifs by X. Given that there are $(L-W+1)$ sub-sequences for each row, the dataset $\mathcal{X}$ of motifs consists of all $(L-W+1)^n$ different combinations of the sub-sequences in Y.

motif X by a PWM f defined as:

$$f(X) \triangleq \begin{bmatrix} f_{1T} & f_{2T} & f_{3T} & \cdots & f_{W-1,T} & f_{WT} \\ f_{1C} & f_{2C} & f_{3C} & \cdots & f_{W-1,C} & f_{WC} \\ f_{1A} & f_{2A} & f_{3A} & \cdots & f_{W-1,A} & f_{WA} \\ f_{1G} & f_{2G} & f_{3G} & \cdots & f_{W-1,G} & f_{WG} \end{bmatrix} \quad (5)$$

where, e.g. f_{jT} denotes the relative frequency of the symbol T in the jth column of the sub-alignment X. The PWM f represents the empirical distribution of DNA bases at each column of X. Each column of a PWM (e.g. f_i) is the set of parameters of a categorical distribution (commonly referred to as the "multinomial distribution" in literature imprecisely) that is defined as a probability distribution that describes the result of the event of randomly selecting one of K possible outcomes (e.g. $K = 4$ for DNA). For sequences of different alphabet, e.g. protein sequences, the PWM is defined with 20 rows corresponding to the number of amino-acid residues.

To quantify the strength or specificity of a motif we use *information content* measure [42][3] that is defined as the divergence of the PWM of a motif from a background distribution. Specifically, for a motif with a PWM f, the divergence from a background distribution g is

defined as the Kullback-Leibler (KL) distance of f and g [41][3] as in the following:

$$D(f,g) \triangleq \sum_{j=1}^{W} \sum_{k\in\{T,C,A,G\}} f_{jk} \log \frac{f_{jk}}{g_k}$$

where f_{jk} is defined in (5) and g_k is the background distribution of base k.

The divergence, $D(f,g)$, also also known as the *biological information content* of the motif [42]. Throughout the manuscript, we refer to a motif X by its PWM f. We also use the strength of a motif, its specificity as well as its information content, interchangeably for the divergence, $D(f,g)$.

4. Bounding the motif p-value

4.1. *Probability of a motif*

We consider a sampling process during which motifs X are drawn from a source according to the background distribution g. In this framework, the event of observing a motif is the *probability of motif* X under the background distribution g and represent it by $P_g(X)$.

Note that in the discussion below we use classical results from information theory. In this theory, the PWM of a motif (equivalently its empirical distribution) is commonly referred to as the *type* of X. The discussion presented here is part of the *Method of Types* [48] that studies statistical properties of sequences based on their types (empirical distributions).

The probability of motif X can be written in terms of its PWM using the following lemma:

Lemma 4.1. *Suppose a motif X is drawn independently and identically (*i.i.d*) according to g and has a PWM (empirical distribution) f (defined in 5). The probability of X under g, denoted by $P_g(X)$ is given by:*

$$P_g(X) = 2^{-n(H(f)+D(f,g))} \tag{6}$$

where H(f) is the binary entropy of f defined as follows:

$$H(f) = -\sum_{j=1}^{W} \sum_{k\in\{T,C,A,G\}} f_{jk} \log f_{jk}$$

and $D(f,g)$ is defined in (3.1).

proof: See ([31], page 281).

□

This lemma gives the probability of a single motif X. In order to compute the probability of a set motifs with a given PWM we need to add up the probabilities of all individual motifs with PWM equal to f as described in the following section.

4.1.1. *Class of a position weight matrix and its probability*

Let us first define the set of all motifs X's that have the same PWM f. This set is commonly referred to as *the class* of the PWM f:

$$T(f) \triangleq \{X \in \mathcal{X} | pwm(X) = f\}, \tag{7}$$

If we count the number of motifs in this class and add their corresponding probabilities using (6) we can compute the probability of all X's with PWM f. For this purpose, we use the following lemma that gives the size of the class of a PWM f:

Lemma 4.2. *The size of the type class of f is upper-bounded as follows:*

$$|T(f)| \leq 2^{nH(f)} \tag{8}$$

proof: See ([31], page 282).

□

It can be seen from (8) that as f changes in such a way that has a larger entropy (e.g. as it gets closer to a uniform distribution as the background distribution g), the total number of motifs, X with PWM f becomes exponentially large. Alternatively, when f is such that its entropy is lower, i.e. it is a highly skewed PWM corresponding to a motif with strong specificity, the number of motifs with a PWM equal to f becomes exponentially small.

Now, we can compute the probability of all motifs with a PWM f by adding the probabilities of all motifs $X \in T(f)$ for each of which we know the probability obtained in (6):

Lemma 4.3. *If motifs X are drawn* i.i.d *according to a distribution g, the probability of motifs that all have a PWM f is upper-bounded as follows:*

$$P_g(T(f)) \leq 2^{-nD(f,g)} \tag{9}$$

proof: The probability of a class $T(f)$ can be written as:

$$\begin{aligned}
P_g(T(f)) &= \sum_{X \in T(f)} P_g(X) \\
&= \sum_{X \in T(f)} 2^{-n(D(f,g)+H(f))} \qquad (10) \\
&= |T(f)| 2^{-n(D(f,g)+H(f))} \\
&\leq 2^{nH(f)} 2^{-n(D(f,g)+H(f))} \qquad (11) \\
&= 2^{-nD(f,g)}
\end{aligned}$$

where in (10) we used (6) of Lemma 4.1 and in (11) we used (8) of Lemma 4.2.

□

According to this lemma, the upper-bound on the probability of all motifs with PWM equal to f is exponentially proportional to the divergence between f and g. Therefore, as f gets closer (in the divergence sense) to g, the probability of observing motifs becomes closer to 1. On the other hand, the probability of strong motifs with large divergence from background, i.e. larger $D(f,g)$, is exponentially small.

So far we have computed the probability of all motifs with a PWM f. We now can compute the p-value of a motif with PWM f. For this purpose, we first determine the number of possible PWMs as described in the following section.

4.1.2. *Number of possible PWMs*

In the following, drive a bound on the number of possible PWMs.

First, let us consider only one column of a PWM as in (5). If there are n sequences in the motif, the column has n symbols chosen from the symbols in $\mathcal{A}$. One can enumerate all possible distributions of symbols in this column:

$$\mathcal{P} = \left\{ (f_1^T, f_1^C, f_1^A, f_1^G) : (\frac{0}{n}, \frac{0}{n}, \frac{0}{n}, \frac{n}{n}), (\frac{0}{n}, \frac{0}{n}, \frac{1}{n}, \frac{n-1}{n}), ..., (\frac{n}{n}, \frac{0}{n}, \frac{0}{n}, \frac{0}{n}) \right\}$$

It can be seen that the numerator of frequencies change from 0 to n. Furthermore, there are three independent frequencies in this PWM, i.e. the last one is fixed by the rest to have a sum equal to 1. Therefore, there are about $(n+1)^3$ different possible arrangements of this frequencies. We formalize this idea for an extended number of columns, W, in the following lemma [31]:

Lemma 4.4. *For a motif of width W, there are at most $|\mathcal{P}| \leq (n+1)^{W(|\mathcal{A}|-1)}$ PWMs in $\mathcal{P}$.*

proof: There are $|\mathcal{A}| - 1$ components in the PWM of any column (the last component is fixed by the the others). The numerator of each component can take $n+1$ values. Therefore, each column can have $(n+1)^{|\mathcal{A}|-1}$ PWMs. Since each column is independently and identically distributed, there are $(n+1)^{W(|\mathcal{A}|-1)}$ different PWMs for the motif of width W.

□

We are now able to compute an upper bound on the p-value of a motif.

4.2. *A bound on the motif the p-value*

Consider a motif X with a PWM, f, with strength $D(f,g)$. The p-value of this motif is defined as the probability of all motifs, X', that have PWMs, h, that are diverged by at least $D(f,g)$:

$$pval(X) \triangleq \sum_{X':D(h,g)\geq D(f,g)} P_g(X') \tag{12}$$

where P_g is as defined previously, and the sum is over motifs X‘ that have PWMs h.

By defining the maximum number of possible PWMs for a motif (of width W) and knowing the probability of the class of each PWM (Lemma 4.3) we can now compute the p-value of a motif with PWM f. The main idea, as explained before, is to first define the set of all motifs with PWMs stronger than f, i.e. with $D \geq D(f,g)$ and then to use Lemma 4.3 to compute its probability. This idea is formalized in the following theorem known as Sanov's Theorem in large-deviation theory. Here we provide a simplified version of the proof that is only applicable to our case. Interested readers are referred to ([31], page 292) for general theorem and technical details.

Lemma 4.5. *Assume a given set of motifs, $\mathcal{X}$, generated according to a background distribution g and a given PWM f that is diverged from the background by $D(f,g)$. The p-value of X defined in (12) is upper bounded by:*

$$pval(X) \leq (n+1)^{W(|\mathcal{A}|-1)} 2^{-nD(f,g)} \tag{13}$$

proof: We denote by $\mathcal{E}(f)$ the set of all motifs, X, that have a PWM h that is diverged from g at least by $D(f,g)$:

$$\mathcal{E}(f) \triangleq \{X \in \mathcal{X} | pwm(X) = h, D(h,g) \geq D(f,g)\}, \tag{14}$$

By definition, the probability of the set $\mathcal{E}$ is the p-value of motif with a PWM f. The probability of the set $\mathcal{E}$ is equal to the sum of the probabilities of the classes of PWMs in $\mathcal{E}$. We have:

$$\begin{aligned}
pval(X) &\triangleq P_g(\mathcal{E}) && (15)\\
&= \sum_{h\in\mathcal{E}} P_g(T(h)) && (16)\\
&\leq \sum_{h\in\mathcal{E}} 2^{-nD(f,g)} && (17)\\
&\leq \sum_{h\in\mathcal{E}} \max_{h\in\mathcal{E}} 2^{-nD(h,g)} && (18)\\
&= \sum_{h\in\mathcal{E}} 2^{-n\min_{h\in\mathcal{E}} D(h,g)} && (19)\\
&\leq \sum_{h\in\mathcal{E}} 2^{-nD(f,g)} && (20)\\
&= 2^{-nD(f,g)} \sum_{h\in\mathcal{E}} (1) && (21)\\
&\leq 2^{-nD(f,g)} (n+1)^{W(|\mathcal{A}|-1)} && (22)
\end{aligned}$$

In (15) we used the fact that, by definition, the probability of the set $\mathcal{E}$ is the sum of probabilities of the classes of PWMs in E. In (17) we used (9) of Lemma 4.3 that gives an upper-bound on the probability of the class of a PWM h. Inequality (18) in valid in we replace all $2^{-nD(h,g)}$ in summation with its maximum value. Similarly, this is valid if we replace its exponent with its minimum in Inequality (19). By definition of the set $\mathcal{E}$ in (14), all its PWMs, i.e. all $h \in \mathcal{E}$ have a divergence not less than $D(f,g)$. Therefore, we have $\min_{h\in\mathcal{E}} D(h,g) = D(f,g)$ in Inequality (20). It can be seen in (21) that $D(f,g)$ is independent of the summation and therefore can be taken out. In (22) we replace the summation with the number of its components, defined by the total number of possible PWMs given by Lemma 4.4.

□

It is important to emphasize that the bound given in (13) is not tight. Note that in (20) we replaced all possible PWMs, h, that have greater divergence than $D(f,g)$ with f. Because of this, we were able to bring the exponential out of the summation in (21) and then replace the total number of PWMs, h, with the maximum number of possible PWMs (i.e. $(n+1)^{W(|\mathcal{A}|-1)}$ Lemma 4.4) to count the summation and add probabilities. Depending on $D(f,g)$ this can be significantly larger than the real number of PWMs that diverge from the background more than $D(f,g)$. Therefore, in these cases, the bound on the p-value is extremely conservative and is significantly larger than the real p-value.

In ideal cases, where an accurate p-value is to be computed, one needs to enumerate all possible PWMs, h, in (21); a problem that becomes exponentially large for practically interesting values of n and W.

This Lemma provides an upper-bound on the p-value of a motif with PWM f presented in Eq. 2.

5. Proof of the main theorem

Theorem 5.1. *Assume a set $\mathcal{Y}$ of n sequences of length L of symbols from an alphabet $|\mathcal{A}|$.*

(a) The expected number of motifs of size $W < L - 1$ with strength equal or greater than $D(f,g)$ observed in $\mathcal{Y}$ is less than 1 when L is smaller than the following bound:

$$L < W - 1 + \frac{2^{D(f,g)}}{(n+1)^{W(|\mathcal{A}|-1)/n}} \tag{23}$$

where $|\mathcal{A}|$ is the cardinality of the set $\mathcal{A}$, e.g. $|\mathcal{A}| = 4$ for DNA sequences with $\mathcal{A} = \{A, T, C, G\}$.

(b) Furthermore, when 1 or more motifs are expected to occur by chance (i.e. false-positives) with strength at least as great as some threshold D^*, i.e. for these motifs $D(f,g) \geq D^*$, an upper bound on the threshold is given by:

$$D^* \leq \log_2 L + (|\mathcal{A}| - 1)W \frac{\log_2(n+1)}{n} \tag{24}$$

Proof. When the probability of motifs with a PWM that is diverged as much as or greater than $D(f,g)$ multiplied by the size of the dataset derived in (4) gives the expected number of such motifs observed in the dataset, denoted by N_f:

$$N_f = |\mathcal{X}| P_g(\mathcal{E}(f))$$

We find an upper-bound for N_f by substituting the upper-bound on $P_g(\mathcal{E}(f))$ given by the Lemma 4.5:

$$\begin{aligned} N_f &= |\mathcal{X}| P_g(\mathcal{E}(f)) \\ &\leq |\mathcal{X}|(n+1)^{W(|\mathcal{A}|-1)} 2^{-nD(f,g)} \\ &= (L - W + 1)^n (n+1)^{W(|\mathcal{A}|-1)} 2^{-nD(f,g)} \end{aligned}$$

Therefore:

$$N_f \leq (L - W + 1)^n (n+1)^{W(|\mathcal{A}|-1)} 2^{-nD(f,g)} \tag{25}$$

(a) By letting the upper-bound on N_f to be less than 1 we have::

$$N_f \leq (L - W + 1)^n (n+1)^{W(|\mathcal{A}|-1)} 2^{-nD(f,g)} < 1$$

which results in:

$$(L - W + 1)^n (n+1)^{W(|\mathcal{A}|-1)} 2^{-nD(f,g)} < 1$$

or:

$$L < W - 1 + \frac{2^{D(f,g)}}{(n+1)^{W(|\mathcal{A}|-1)/n}} \tag{26}$$

Therefore, if this inequality holds we have $N_f < 1$ and part (a) is proved.

(b) Let N_{D^*} be the number of motifs expected to be observed by chance (i.e. false positives) in $\mathcal{Y}$ with strength greater than the threshold D^*. We set the arbitrary threshold D^* to assume

values equal to the strength of false-positives, i.e. $D^* = D(f, g)$. In that case, by definition, we have $N_{D^*} = N_f$, and therefore from (25) we have::

$$(L - W + 1)^n (n + 1)^{W(|\mathcal{A}|-1)} 2^{-nD^*} \geq N_{D^*} \geq 1$$

which results in:

$$(L - W + 1)^n (n + 1)^{W(|\mathcal{A}|-1)} 2^{-nD^*} \geq 1$$

or:

$$L \geq W - 1 + \frac{2^{D^*}}{(n+1)^{W(|\mathcal{A}|-1)/n}} \tag{27}$$

$$\geq \frac{2^{D^*}}{(n+1)^{W(|\mathcal{A}|-1)/n}}. \tag{28}$$

By taking $\log_2$ from both sides of the inequality part (b) is proved. □

Part (a) of this theorem gives an upper-bound on the size of the dataset, which if satisfied, no false-positive is expected to be observed in the dataset. Alternatively, given a dataset of n sequences with length L, Part (b) provides an upper-bound on the strength of possible false-positive motifs with width W.
