# Supplementary material for: Towards a theoretical understanding of false positives in DNA motif finding
Source: BMC Bioinformatics. 2012 Jun 27;13:151. doi: 10.1186/1471-2105-13-151 (PMC3436861; doi:10.1186/1471-2105-13-151)
Supplement: Additional file 2 — Figure S1. The comparison between bound on the p-value (Eq. 2) and the p-value computed by an FFT-based method. Figure S2. Theoretical bound on sequence length compared with MEME results. Figure S3. Theoretical bound on sequence length compared with GIMSAN results. (PDF 225 kb) [file 1471-2105-13-151-S2.pdf]

Supplementary figures

Towards a theoretical understanding of false positives in DNA motif finding

Amin Zia, Alan M. Moses

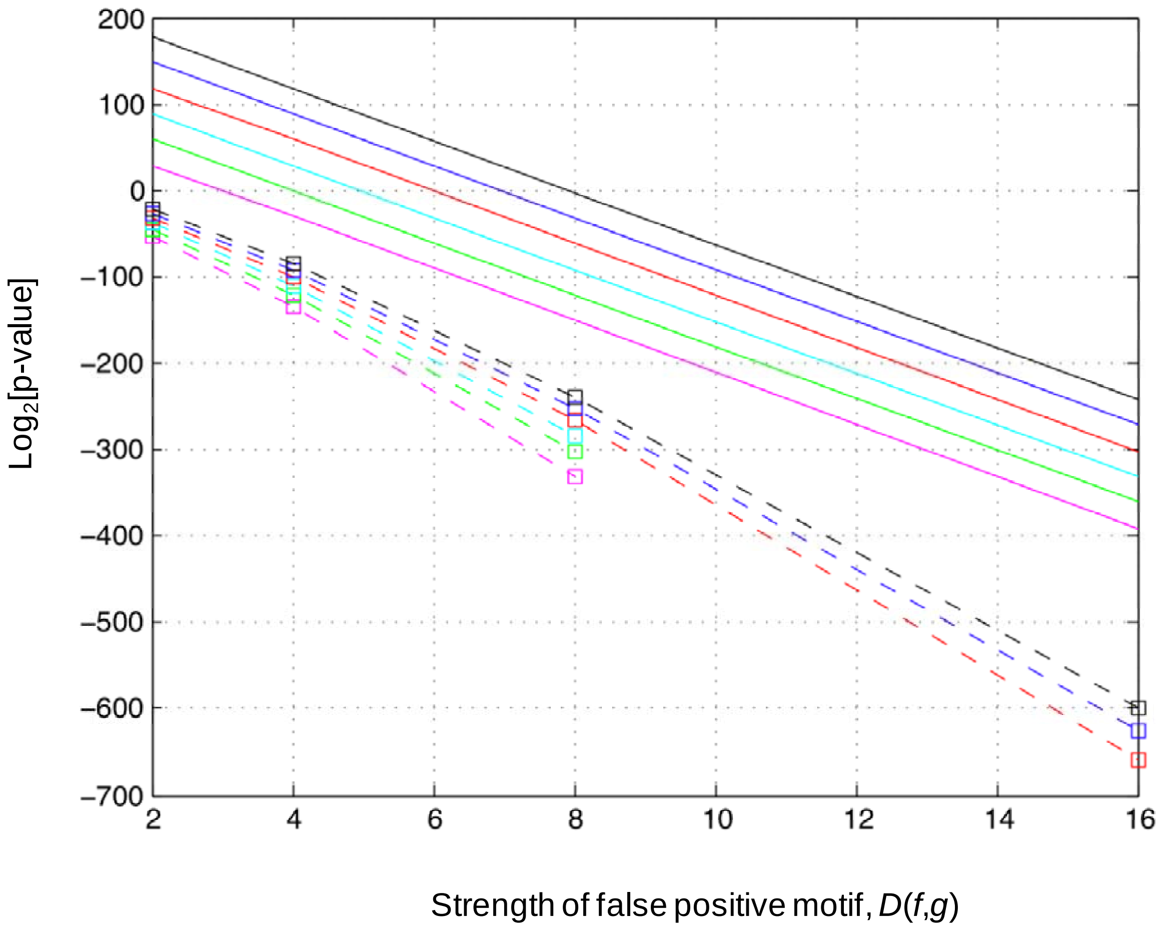

Figure S-1. The comparison between bound on the p-value (Eq. 2) and the p-value computed by an FFT-based method.

The number of sequences in the dataset is fixed to n=30 and for each motif strength of $D(f,g)$={2,4,8,16} bits, the p-value is computed using Eq. 2 (solid traces) and an FFT-based method (dashed traces and squares, [27]). Black, Blue, Red, Cyan, Green and Magenta traces and squares indicate motifs of widths W= 6,8,10,12 and 14.

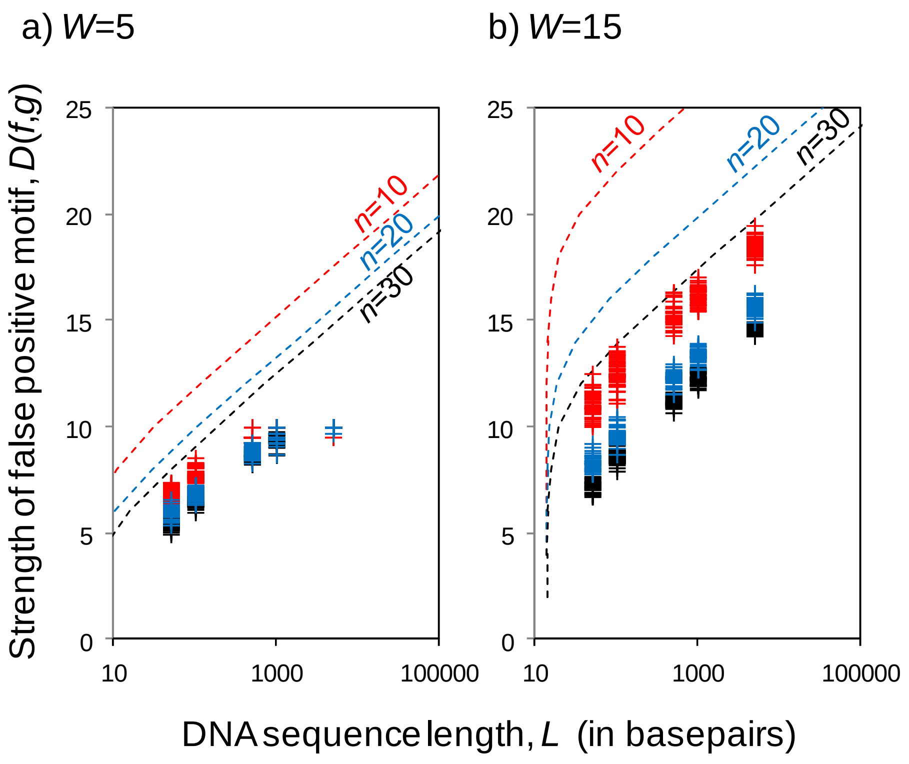

Figure S-2. Theoretical bound on sequence length compared with MEME results

Theoretical bound on sequence length, *L*, at which less than one false-positive motifs with information content *D*(*f*,*g*) is expected (dashed lines) compared to experimental results of MEME (crosses) for motif width (a) *W*=5 or (b) *W*=15. The region of the plot above the traces represents the parameter space in which less than one false positive is expected. The black, red and blue results are for three different numbers of sequences, *n*={10,20,30}, respectively. Each cross represents the motif identified in one of 50 random sequence sets.

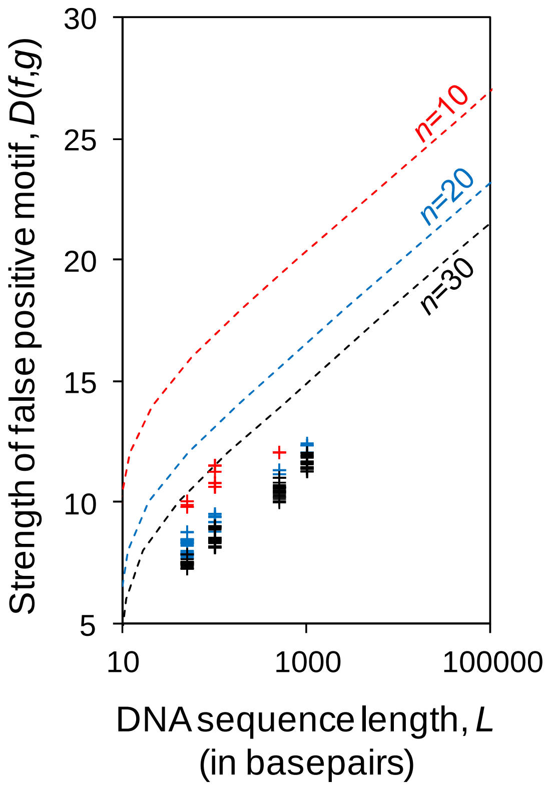

Figure S-3. Theoretical bound on sequence length compared with GIMSAN results

Theoretical bound on sequence length, *L*, at which less than one false-positive motifs with information content *D*(*f*,*g*) is expected (dashed lines) compared to experimental results of GIMSAN (crosses) for motif width *W*=10. The region of the plot above the traces represents the parameter space in which less than one false positive is expected. The red, blue and black traces are for three different numbers of sequences, *n*={10,20,30}. Each cross represents a motif identified with p≤0.01 in a random sequence set.
